# Standard method for microCT-based additive manufacturing quality control 4: Metal powder analysis

**DOI:** 10.1016/j.mex.2018.10.021

**Published:** 2018-10-23

**Authors:** Anton du Plessis, Philip Sperling, Andre Beerlink, Willie B. du Preez, Stephan G. le Roux

**Affiliations:** a CT Scanner Facility, Stellenbosch University, Stellenbosch, South Africa; b YXLON International GmbH, Hamburg, Germany; c Dept of Mechanical Engineering Dept, Central University of Technology, Free State, South Africa

**Keywords:** Standard method for microCT-based additive manufacturing quality control 4: metal powder analysis, Additive manufacturing, MicroCT, X-ray, Tomography, Non-destructive testing, Powder, Particle, Characterization

## Abstract

X-ray micro computed tomography (microCT) can be applied to analyse powder feedstock used in additive manufacturing. In this paper, we demonstrate a dedicated workflow for this analysis method, specifically for Ti6Al4V powder typically used in commercial powder bed fusion (PBF) additive manufacturing (AM) systems. The methodology presented includes sample size requirements, scan conditions and settings, reconstruction and image analysis procedures. We envisage this method will support standardization in powder analysis in the additive manufacturing community. This is aimed at ultimately improving the quality of additively manufactured parts, through the identification of impurities and defects in powders.

• MicroCT analysis of metal powders for additive manufacturing
• Method describes a standard workflow simplifying usage of the technique
• Sample requirements and image analysis workflow is described

## Graphical abstract

**Figure:**
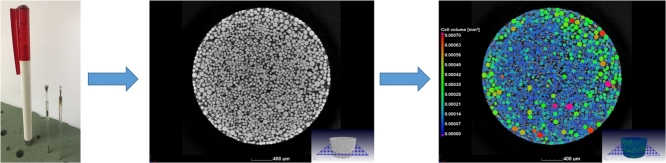

**Specifications Table**

**Table:** 

Subject Area	Engineering
More specific subject area:	Additive manufacturing / advanced manufacturing / mechanical & industrial engineering
Method name:	MicroCT analysis of metal powder - standard method
Name and reference of original method	None yet, only isolated cases of individual researchers who have done slight variations of the technique, as cited in the paper
Resource availability	All described in paper already with references: typical micro/nanoCT scanner, 3D image analysis software eg. Volume Graphics VGStudio Max 3.2

## Method details

Powder analysis is traditionally done using a laser diffraction method, such as described in ASTM B822 – 17. This laser diffraction method is simple, fast and provides estimated particle size distributions; however this is based on a spherical particle assumption. Often the particles found may be significantly non-spherical. Some studies have also made use of microscopy and image analysis to analyse the morphology of intact metal powder particles. However, the microscopy method can only provide pseudo-3D images, not true 3D images, hence only qualitative analysis is possible and internal porosity inside particles cannot be visualized. Sectioning of particles embedded in resin and imaging of these particles using a microscope is possible and has been used in combination with stereological image analysis to provide particle size distributions and shape information and in this case internal porosity may be visualized. However, this method has the disadvantage of being very time consuming and statistically challenging to calculate proper particle sizes, due to the sectioning of particles being inherently not through the middle in most cases. Another disadvantage with sectioning is that the sectioning process may smear over small pores and may therefore affect the images obtained.

Metal powder analysis in AM has been applied to monitor changes in powder quality upon many cycles of re-use [1]. In this study it was shown that powder particles become less spherical and have an increasingly rougher surface with an increasing number of re-use cycles. It may also be that other types of powder partially fuse which can decrease powder bed flowability properties, but this has not been directly reported in the scientific literature to our knowledge. In any case the quality of re-used powder needs characterization to ensure maintenance of optimal properties.

It has been demonstrated that porosity inside powders may be transferred to the meltpool and hence to the final part [2], in a synchrotron tomography study. It is also known that the particle size distribution and the sphericity of the particles affect the flowability of the powders, which in turn affects the powder bed quality in terms of spreading and packing density.

The use of X-ray CT for analysis of particle shapes was originally demonstrated as early as 2002 [3] and more recently the method was compared with various other methods for metal powder analysis for additive manufacturing [4]. This work demonstrated the advantage of CT and also discussed the effect of recycling of powder. The use of laboratory microCT for imaging of small particles such as metal powders was demonstrated in a few more studies recently, using different procedures and sample preparation. In one such study the interest was simply to visualize porosity in powders, without discussion of the procedure used [5]. In a study of the particle shapes of smaller very irregular particles in the range 50–150 μm, it was shown that microCT can be applied successfully to characterize the particle shapes [6]. In another paper a methodology was described for microCT scans up to 3 μm resolution, with a dedicated image analysis procedure [7]. In this study, the particles were embedded in resin and the resin machined to a rod geometry. This allows stability and ease of mounting of the sample in the microCT instrument for high magnification. The same authors more recently extended this work to smaller powders and scan resolution down to 0.7 μm [8]. This paper describes a workflow for obtaining powder porosity by microCT but the description for obtaining particle size distribution is not clear, and the procedure makes use of user-dependant procedures for de-noising and thresholding. Nevertheless, it demonstrates feasibility of the method and applicability to characterization of powders typically used in AM, and does provide a first step towards standardization. All the above-mentioned studies make use of carefully mounted particles in resin. This procedure of sample preparation is time consuming and limits the wider uptake of this method.

In the method presented here, we demonstrate a simplified methodology where no sample preparation is required: the particles are loaded in a small cup or tube and scanned at 0.7–1.5 μm resolution (depending on the particle sizes expected), for a total scan time of approximately 2–3 h per sample. Depending on the analysis required the image analysis procedure involves roughly the same time investment as scanning time, which can allow optimized workflow for large numbers of samples (image analysis of first sample done during scan of second sample, etc). We have applied a simplified version of this method recently to the analysis of heavy mineral sands, as shown in [9]. The procedures described here in detail requires high resolution scanning possible with any system containing an X-ray source and associated hardware allowing nanoCT, ie. submicron source spot size and system stability. The method also uses image analysis routines available in commercial software, which removes potential human bias from the methodology. Such simplified unbiased methods are important to the proper use of the technology to support the additive manufacturing community, and is one of a number of standardized methods developed in our group [[10], [11], [12]] and mentioned in a recent review of the technology applied to AM [13]. As described in Seifi et al [14], there is currently an urgent need for standardization in the AM community and the quality inspection of metal powders is part of this requirement.

## The method

X-ray micro computed tomography [15] was used in this study using optimization procedures as described in [16]. Metal powder was acquired from a recent study of powders used in different commercial systems [17], with the two demonstrated here originating from commercial supplier TLS Technik GmbH with large size fraction (LENS powder, 40–100 μm) and the other with small particle size distribution (<40 μm) for a DMLS AM machine. These cover the typical size ranges of powders in use commercially in AM systems. The methodology is demonstrated for the larger powder, while the smaller powder is shown in the last figure and in the associated image analysis workflow video (Supplementary material).

**Fig. 1:**
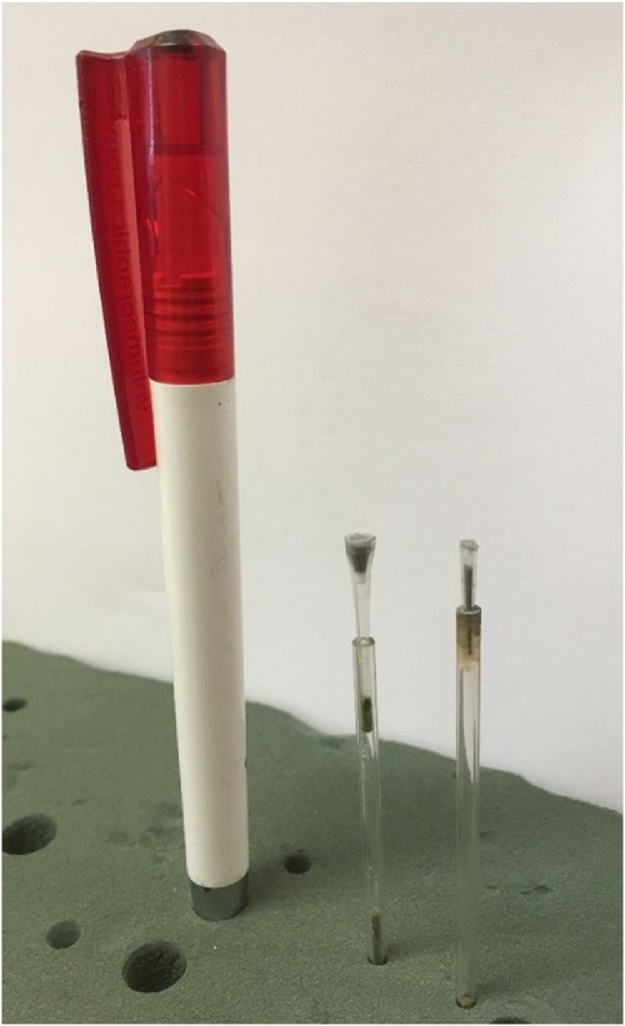
Sample mounting – the pen is for scale indication, the powder is loaded in the cup or tube as shown – sample on left is for 1.5 μm scan, sample on right is for smaller powders for 0.7 μm scan.

Samples were loaded into a plastic cup (for the larger powder) or a thin plastic tube (for the smaller powder), this was fixed on a glass rod and mounted as close as possible to the X-ray source; the sample mounts containing powders are shown in Fig. 1. This allows, with high quality parameters and reasonable scan times, voxel sizes of 1.5 μm for the cup and 0.7 μm for the tube. The larger powder cup has a total width of metal powder of approx. 2.5 mm, and this requires a 0.1 mm copper filter to prevent beam hardening artefacts. The scan settings are with an X-ray spot size approximately 2 μm. For the smaller container with total powder width of 0.7 mm, no beam filter was necessary. In this latter case the X-ray spot is kept below 0.9 μm using suitable apertures (system specific). In both cases the beam hardening correction applied was very strong to ensure no greyscale variation across the diameter of the cup, which can affect the segmentation step. The scan parameters used are shown in Table 1. The best contrast is obtained when the entire sample width fits the field of view, the current is increased to the maximum allowed for the X-ray spot size (usually system controlled limits), and the noise is limited by keeping the detector as close as possible to the source. This means that the sample is very close to the source, which requires a very precise glass rod with no excess material which can limit the rotation (see Fig. 1). Scan settings include detector shift, to remove possible ring artefacts and averaging of 2 images at each step position while the first image at each step position is discarded. A full rotation is completed with up to 3000 step positions. The powder must settle in the container so it is suggested to run a dummy scan prior to the real scan, this also allows the system to thermally stabilize and limits X-ray spot drift. It should be mentioned that 10 μm resolution scans of powder have been suggested in at least one aerospace quality control guideline, to ensure no impurities are present in the powder. Such a scan does not resolve powder particles but can be very fast and can therefore be additionally done prior to higher resolution scans. Such fast scans will immediately indicate the presence of dense impurities but more detailed images are required for porosity analysis or further analysis as described in this paper.

**Table 1:** Scan settings for each type of scan.

Voxel size (μm)	Voltage (kV)	Current (μA)	Scan time (hrs)	Field of view (mm)
2	100	100	2	2.5
0.7	100	280, with apertures	3	0.7

**Fig. 2:**
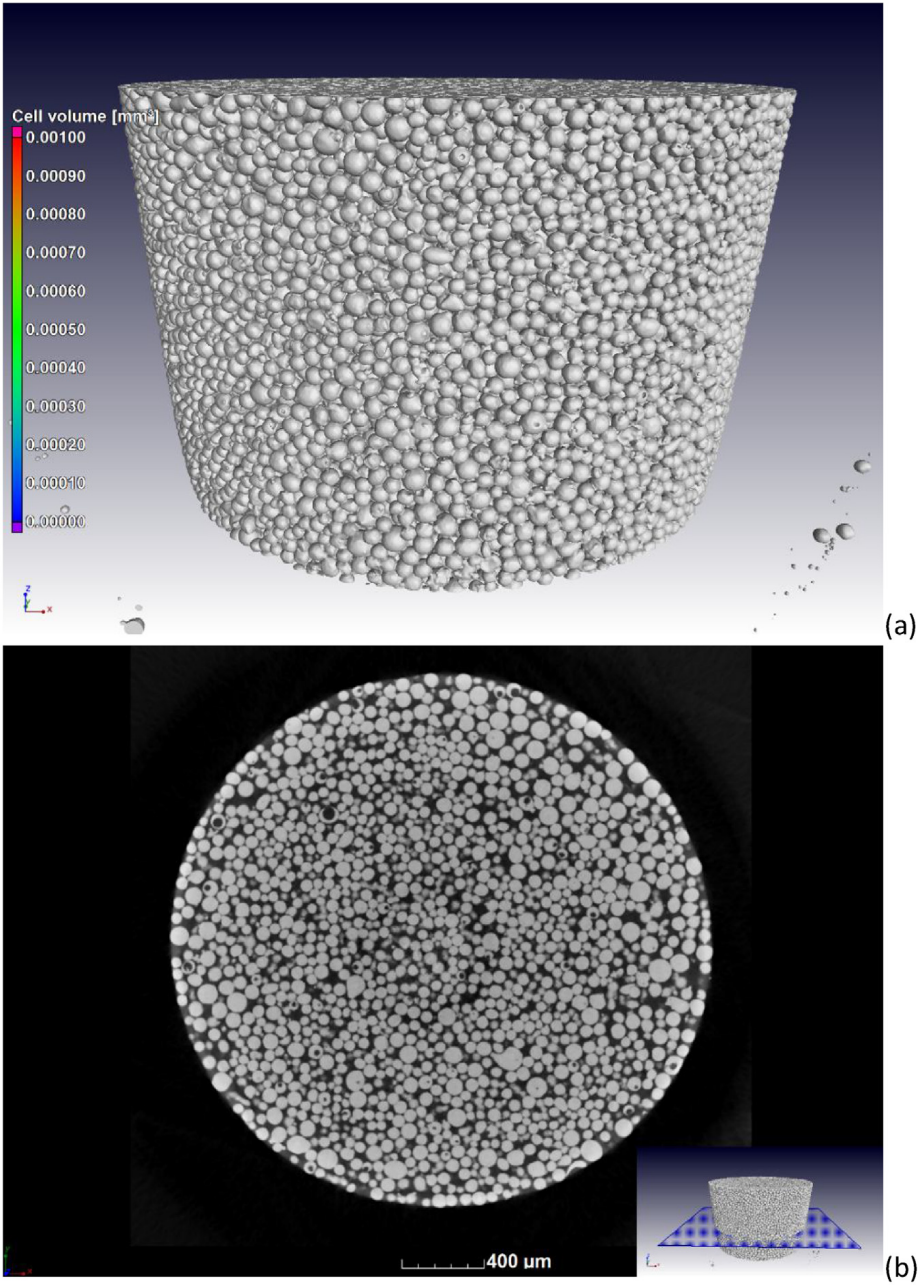
CT scan results showing (a) 3D surface view and (b) CT slice image clearly indicating particles with pore spaces (black circles). Visualizations performed with VGStudio MAX 3.1.

Following a good quality microCT scan at the parameters in Table 1, reconstruction using a strong beam hardening correction and de-noising using a default adaptive Gauss filter in VGStudio MAX 3.1 (Volume Graphics, Heidelberg, Germany), the resulting microCT slice images for the large particle powder are shown in Fig. 2. More details can be seen in the steps shown in the supplementary videos. The 3D image shows the exterior morphology of the particles while the slice images show internal porosity and more details of the morphology. This image can be used to assess the presence of impurities, without any further complex analysis.

**Fig. 3:**
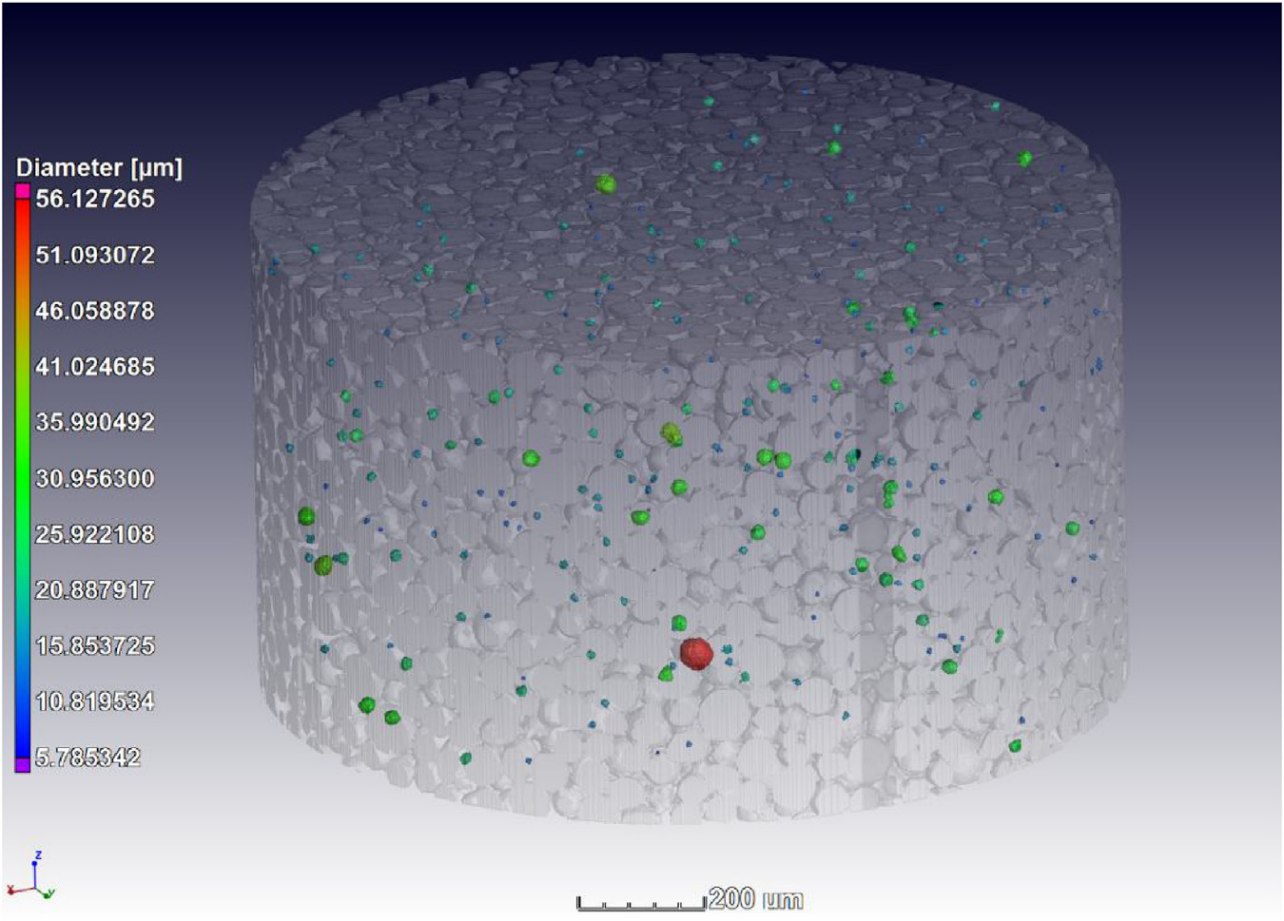
Porosity analysis of powders (closed pores only).

Evaluation of porosity in the powders can be challenging as some pores might be open to the surface of the particle while others are not. The suggestion is to manually evaluate the porosity in slice images. A more quantitative (optional) assessment is described here – this involves selecting the closed porosity only. This can then be used to assess manually the extent of open porosity vs closed porosity. The segmentation method involves applying an advanced surface determination (using the auto function, no human bias) with and without “remove all voids”. In each case an ROI is selected from the surface determination, and the two ROIs are subtracted from one another to leave only the internal pores as an ROI. This ROI is used in a custom defect mask porosity analysis, to provide colour coded porosity information of the closed pores as shown in Fig. 3.

**Fig. 4:**
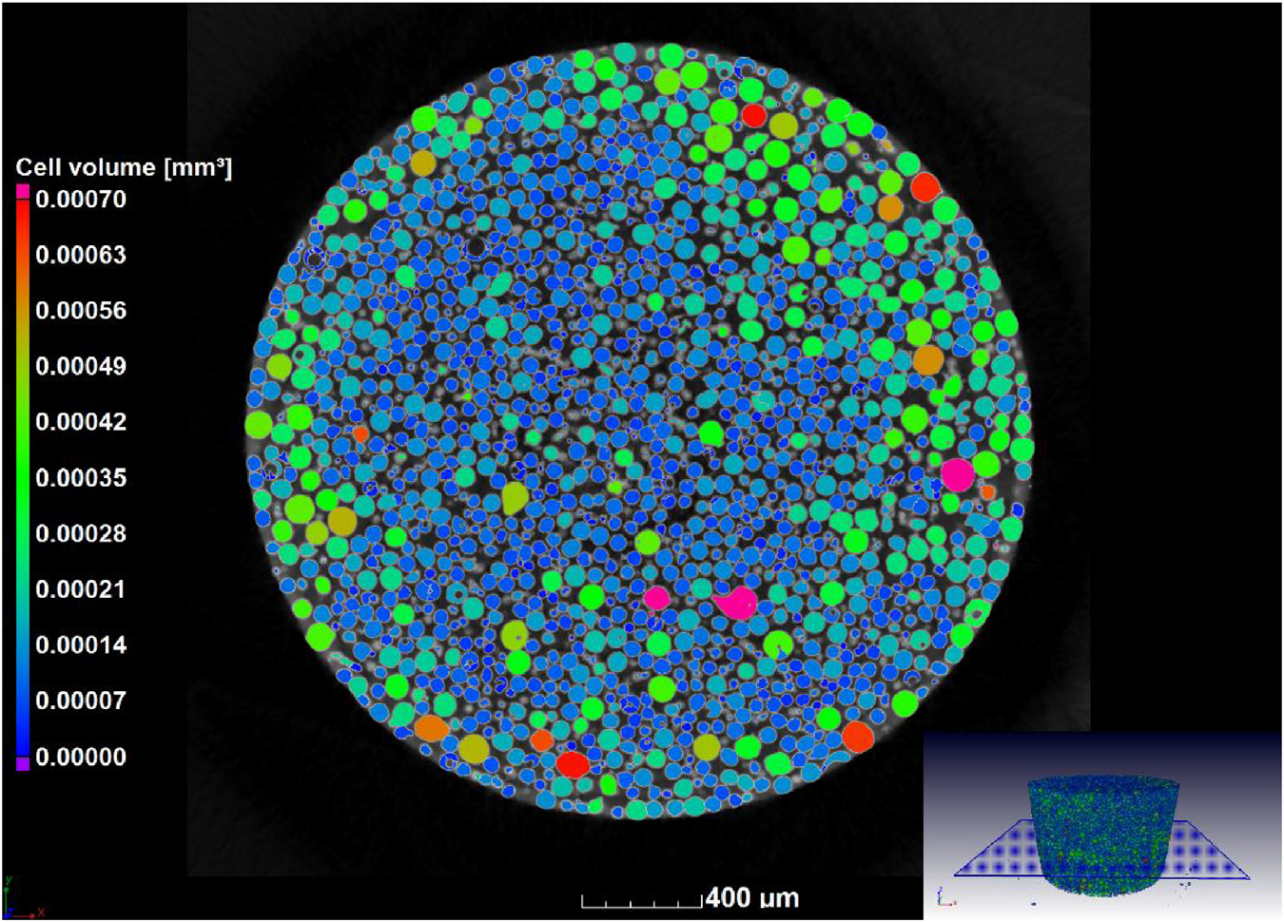
Particle size analysis - colour coding based on individual volumes.

**Fig. 5:**
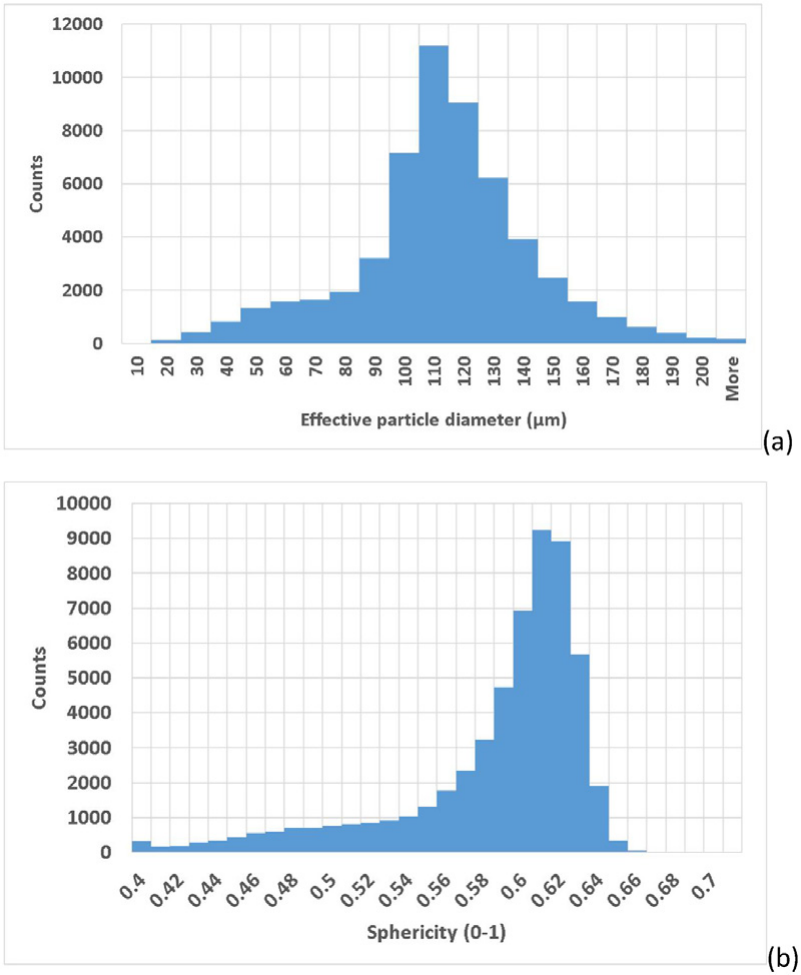
Statistical information obtained by microCT of (a) particle size distribution and (b) sphericity distribution – a total of 62,137 particles were analysed in this data set.

The next step is for analysis of the particle sizes and shapes, for which the VGStudio MAX 3.1 foam structure analysis module is used, applying the algorithm to the material. Default settings are applied to obtain the analysis as shown in Fig. 4, which provides for each particle a volume as shown in the colour coding. The particle size distribution can therefore be analysed in detail (Fig. 5a), as well as the sphericity distribution (Fig. 5b). Sphericity is here defined as the ratio of the surface area of a sphere with the same volume as the particle, relative to the surface area of the particle itself. For statistical analysis the data for each particle is extracted in a CSV file as a spreadsheet. There is no segmentation step, such as typical watershed algorithm used in other software tools, but the splitting of touching particles is affected by a “merge threshold” value which is by default set to 5% and works well in most cases. When it is observed that too much or too little splitting occurs, this value can be adjusted, and this will depend on the scan quality and resolution relative to particle size.

**Fig. 6:**
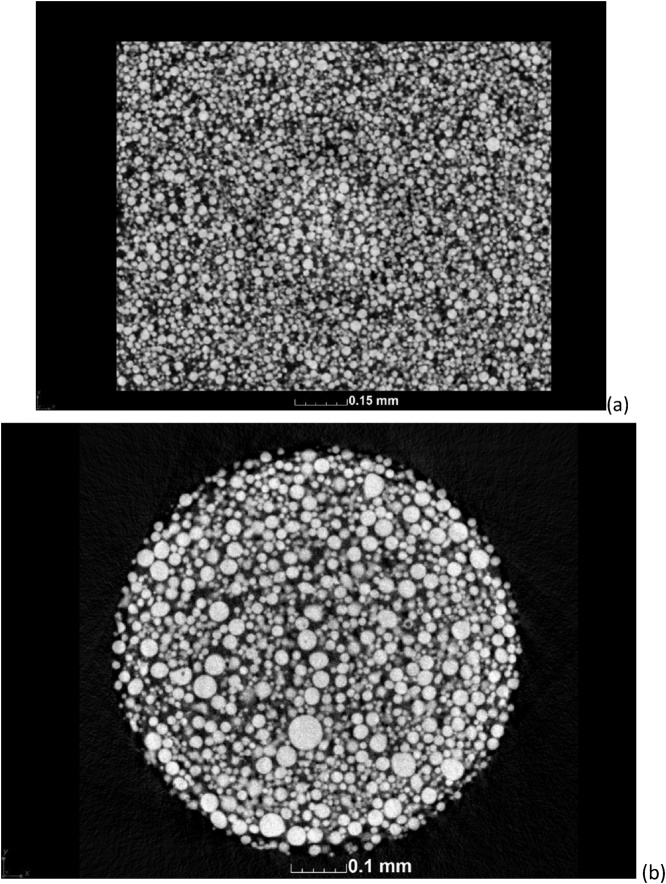
CT slice image of EOS powder with peak of 40 μm at (a) scan resolution 1 μm using the larger container, and an improved scan using a smaller container at (b) 0.7 μm. The smaller sample size requires a smaller container which results in improved scan quality.

**Fig. 7:**
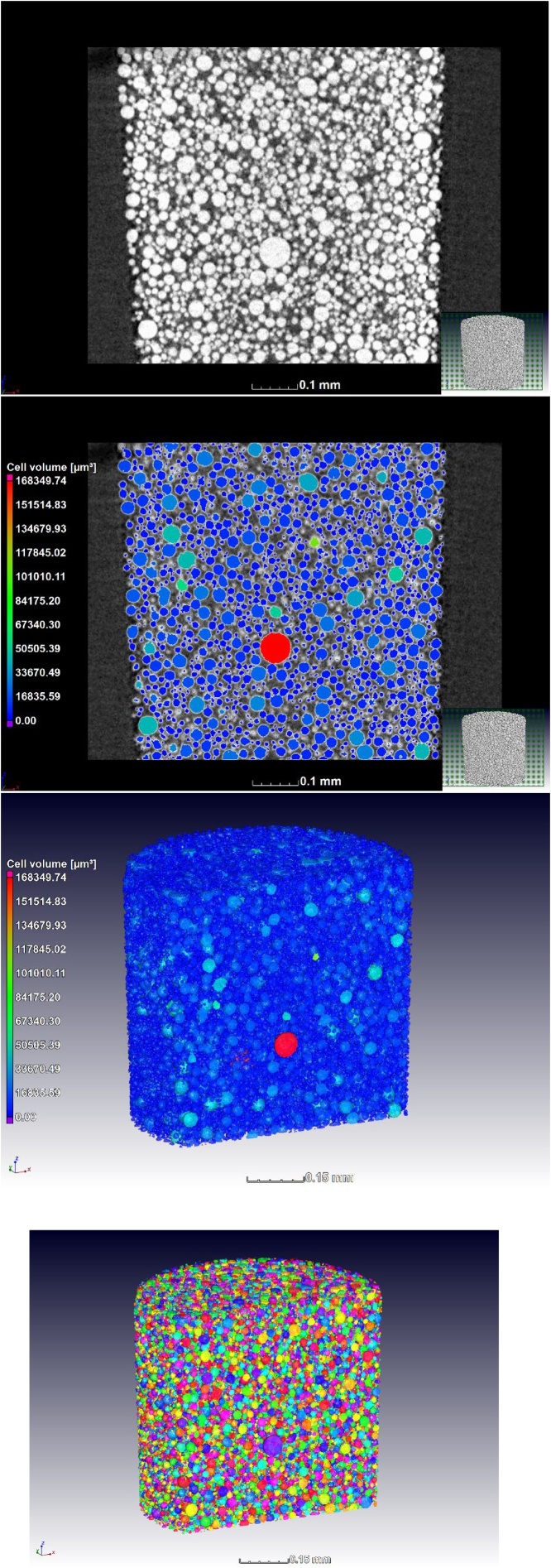
Submicron CT scan of DMLS powder (<40 μm specification). This series shows the slice image without and with analysis, a 3D view of the analysis for size, and a 3D view of the uncoded particles – colours a varied between adjacent particles to highlight the large number of particles.

The above-mentioned example clearly shows what is possible, when the scan resolution is 1.5 μm and the particle size distribution peak is around 100 μm, therefore based on this scan an average of 66 voxels are required across a mean particle for the above detailed analysis. Not only the resolution but also the contrast is important here. In cases when the powder is smaller, this may result in poor contrast and hence the detailed analyses are not possible. In this case impurities can still be checked and estimates can be made of the morphology of the powders. One example of this is shown in Fig. 6a, where the scan resolution of 1 μm for powder with an expected peak of <40 μm is shown. Thesmall size of the powder limited the scan quality and hence limits the further processing of the data when scanned using the 2.5 mm wide cup. Besides resolution, there is also poor penetration and sub-volume scanning, reducing the data quality. The best contrast is found when the entire width of the sample fits in the field of view. A smaller field of view allows more penetration and hence better contrast. Fig. 6b is the result of an improved scan of the same powder using a smaller tube and a higher scan resolution at 0.7 μm for the same powder. The latter scan at 0.7 μm, allowed quantitative analysis as shown in Fig. 7. The images in Fig. 7 demonstrate that the quantitative analyses described can be applied to smaller powders in the same way as described above for larger powders. Though not the topic of investigation of this method description, this smaller powder was measured as having a mean particle diameter of only 14 μm.

This method was recently applied to virgin Ti6Al4V powder as part of a round robin study, and interesting “powder inside powder” was observed for the first time to our knowledge, this is shown in [18].

## Conclusion

A simple method was described which allows high resolution microCT scans and detailed analysis of Ti6Al4V metal powder, typical for laser powder bed fusion systems. While the method described here is for Ti6Al4V particles, it may be modified slightly and applied in a similar manner for lower or higher density particles, and for particles with smaller or larger size distributions. Smaller particles will require a higher resolution scan and potentially a smaller sample tube. For larger particles, a larger field of view is required and larger cup, to ensure no particles are cut off at their edges. Denser particles might require longer scan times and the smallest detected particles might be larger due to increased power required which increases the X-ray spot size.

As a standard method, it is suggested that when an unknown powder is to be tested, the first scan is done at 1.5 μm as described above, which allows to roughly check for impurities, morphology and porosity. If the powder is large enough (approx. > 100 μm), quantitative analysis is also possible with this data, as demonstrated. If quantitative analysis is required but the powder is found to be too small for a clear segmentation, a higher resolution scan is suggested using a smaller tube as shown for 0.7 μm. The examples shown here cover the range of sizes expected for most metal powder bed fusion systems and can therefore be used for this application directly without further modification of the parameters. In this case, powder with mean size of 14 μm was successfully analysed in detail using the 0.7 μm scan settings.

This image analysis methodology provides information on internal porosity (open or closed), particle morphology (volume, surface area, sphericity) and on the presence of impurities such as denser particles. However, the method does not provide information on oxygen content, which is an issue in re-used or exposed powders, and it does not provide information on particles or pores smaller than the voxel size. It also does not necessarily provide information on multi-particles, but this might be an interesting topic for further investigation. It should therefore be used as part of a holistic quality inspection, also incorporating other methods.
